# The third way of inclusive growth in China

**DOI:** 10.1007/s44216-022-00007-5

**Published:** 2022-10-18

**Authors:** Yu Zheng

**Affiliations:** School of International Relations and Public Affairs, Fudan University, Shanghai, China

**Keywords:** Inclusive growth, Poverty alleviation, Common prosperity, Developmental state, Welfare state

## Abstract

In much of the 20th century, the welfare state was regarded as the fundamental means for Western countries to embrace economic liberalization through domestic social contracts, whereas the developmental state was credited for East Asian economies’ growth with equality. However, economic globalization and technological changes have posed serious challenges for both models with respect to containing increasing inequality and achieving inclusive growth. China’s performance in inclusive growth has demonstrated distinct features that differ from the approaches of Western welfare states and East Asian developmental states. China has relied less on conventional means of redistribution, such as taxes and transfers. Instead, it has combined growth-oriented industrial policies, public infrastructure investment, and state-mediated poverty alleviation programs. China’s development strategy reflects a “third way” perspective on inclusive growth that might be instructive for latecomer economies.

China’s recent efforts to narrow inequality in pursuit of the ultimate goal of “common prosperity” have attracted attention and speculation. The Chinese government has promoted policies to increase the incomes of the poor while curbing excessive incomes and banning illegal income among the wealthy. Beijing has also encouraged more charity and donations from profitable firms via the so-called “third distribution,” a type of income distribution based on moral beliefs (Li Yining 2015). Han Wenxiu, an official with the CCP Central Committee for Financial and Economic Affairs, emphasized the need to promote high-quality development: “While the issue of distribution is critical, our goal for common prosperity shall not rely merely on distribution” (Zhang 2021).

Nevertheless, the emphasis on redistribution did not come as a sudden policy change. In 2013, the Chinese government emphasized the need for “forming a reasonable and orderly pattern of income distribution, instituting a fairer and more sustainable social security system, and deepening reform in medicine and health care.” (Communiqué of the Third Plenary Session of the 18th Central Committee of the Communist Party of China n.d.) Shortly thereafter, a major anti-poverty campaign was launched with the goal of lifting the entire Chinese population over the absolute poverty threshold of RMB 2300 in annual income per capita. In February 2021, China officially declared this goal to be accomplished.

Does this drive to common prosperity indicate a transformation of state-society relations from those of a developmental state that prioritizes economic growth to those of a welfare state that emphasizes redistribution? Globalization and technological advances have led to increasing economic inequality and political polarization in many countries. The conventional models of the welfare state and the developmental state face the challenge of mitigating the tension between growth and redistribution. Both models must adapt to the changing environment, albeit in different ways.

We argue that China’s development strategy differs significantly from the approaches of both the conventional welfare state and the developmental state. China’s progress in inclusive growth can be attributed to three factors. First, it implements industrial policy on a massive scale to achieve industrial upgrading. Second, it relies on public infrastructure investment to counter economic slowdowns and external shocks. Third, it employs a state-mediated approach and mobilizes resources for poverty reduction.

China’s approach toward inclusive growth is instructive for other developing countries, particularly latecomers with low starting points. One important point is that while achieving inclusive growth requires developing countries to develop redistributive capacity to address economic inequality, they should not necessarily focus on postproduction redistribution. Rather, countries could act in a more interventionist manner to directly tackle absolute poverty and thus to generate momentum for sustainable development.

This paper proceeds as follows. It first reviews the evolution of the welfare state in Western countries and that of the developmental state in East Asian economies. It then compares the performance on inequality and inclusive growth between different regions. Third, it discusses the challenges Western countries have faced in addressing inequality. Fourth, it discusses East Asian economies’ advantages in containing inequality. Fifth, it analyzes China’s strategy for inclusive growth. Finally, it discusses the implications of the Chinese experience for other developing countries.

## Evolution of the welfare state and the developmental state

In his classic study *The Great Transformation*, Karl Polanyi explains that the failure of the first era of globalization was caused by the expansion of market forces undermining the social structure and giving rise to radical political forces. Therefore, it is necessary to strengthen the state capacity to hinder the disembedding of markets from society. In the post-WWII era, two different models emerged as countries established domestic social contracts while engaging in global markets.

Embedded liberalism, a compromise between laissez-faire liberalism and economic nationalism, provided the framework for understanding the nature of state-society relations in developed countries. “Unlike the economic nationalism of the thirties, it would be multilateral in character; unlike the liberalism of the gold standard and free trade, its multilateralism would be predicated upon domestic interventionism” (Ruggie 1982). Trade and capital liberalization were embedded within social policies designed to compensate those harmed domestically by these policies. The core principle of embedded liberalism is the need to legitimize international markets by reconciling them with social values and shared institutional practices (Abdelal and Ruggie 2009).

The framework of embedded liberalism thus justified the expansion of social protection as compensation for the losers in the global economic engagement of developed countries. A high level of openness accompanied by a large-scale provision of social insurance to reduce social risk has been the defining characteristic of postwar developed countries. In Europe, such social insurance has often taken the form of generous welfare states, while in the United States, more targeted compensation programs, such as Trade Adjustment Assistance (TAA), provide protection against the damaging impact of foreign trade (Aho and Bayard 1984).

While embedded liberalism was prevalent in Western countries, embedded autonomy was the defining feature of East Asian developmental states in response to economic openness (Evans 1995, Bardhan 2016). Emphasizing the importance of maintaining state autonomy while building dense ties to business elites in policy-making, embedded autonomy has been credited for East Asian economies’ successful state involvement in industrial transformation. It also provides an underlying structural basis for balancing growth and distribution. Social policy regimes in East Asia are distinct from Western welfare regimes in their productive intent, in that the latter all prioritize economic growth over social protection (Holiday 2000).

In the report “East Asia Miracle,” the World Bank emphasized two pillars—labor-intensive growth and the accumulation of basic human capital—while placing limited emphasis on a third pillar—social protection—for growth and poverty reduction (World Bank 1993). In particular, education was regarded as an important contributor to East Asia’s equitable income distribution. East Asian governments have focused spending on primary and secondary education and left demand for tertiary education to be largely met by a self-financed private system (Page 1994).

Despite East Asian economies’ minimalist approach to the provision of social protection, they have performed remarkably in alleviating poverty and reducing income inequality compared to other developing regions at a comparative income level (World Bank 2018). Between 1965 and 1996, for example, South Korea reduced its poverty rate from 41.1% to 9.6%, and Thailand’s poverty rate decreased from 57.0% to 11.4% (Quibria 2002).

Since the 1980s, economic globalization and technological advances have increased the gaps between the supply of and demand for redistribution in both developed and developing countries. Both Western countries and East Asian economies have had to adapt to changing international and domestic dynamics, including global volatility, economic inequality, and political polarization.

Western countries have implemented so-called “third way” welfare state reforms, emphasizing the balance between economic security and competitiveness. While these employment-oriented reforms attempted to “transcend both old-style social democracy and neoliberalism,“ (Giddens 1998) the influences of globalization and technological change on inequality seemed to be inevitable.

Driven by the third wave of democratic transition, East Asian economies also conducted welfare reform in the 1990s, shifting from productive welfare strategies and placing more emphasis on redistribution. Since the reforms were instituted under highly favorable economic conditions, these economies could expand welfare entitlements to voter-beneficiaries while increasing international economic integration (Haggard and Kaufman 2008). This strategy of state-mediated international economic integration has also been referred to as embedded neoliberalism (Kurtz and Brooks 2008).

Globalization has in many ways increased the need for the modern welfare state but at the same time reduced the state’s capacity to meet this need. Since the 1990s, both developed and developing countries have experienced deindustrialization, as indicated by the decrease in industrial output and employment as a share of national production and employment. In particular, the premature deindustrialization trend that occurred in both middle-income Latin American countries and low-income African countries is particularly worrisome (Rodrik 2016). This trend is often accompanied by “premature urbanization.” Instead of entering the more productive manufacturing sector, surplus labor from agriculture ends up in the less productive services sector. The benefits of industrialization-driven growth do not trickle down in the form of jobs and other opportunities, leaving the poor population more vulnerable.

Digital technological advances have also contributed to the increase in income inequality. In history, technological advances have played different roles in shaping income distribution. The key technological advances of the nineteenth century replaced skilled workers with new physical capital, raw materials, and unskilled labor. Such developments created a vast demand for unskilled workers and increased the ease with which industrial workers could shift between manufacturing industries, which led to an upward trend of interindustry factor mobility and a downward trend in income inequality (Hiscox 2001). Since the end of the 20th century, however, technological advances have had different effects on income distribution. Investment in information and communication technology (ICT) has required high-skilled workers at the cost of low-skilled ones. In developed countries, growth in the demand for human capital has resulted in the polarization of labor markets, as low-skill workers are replaced by new technologies. ICT could also increase inequality by strengthening the bargaining power of large, financially strong and politically influential corporations (Cerra et al. 2021).

## Comparative performance of inequality and inclusive growth

Inequality has increased in nearly all developed countries, generating profoundly negative economic, social, and political consequences. As shown in Table 1, between 1980 and 2019, the Gini coefficient in the US increased from 0.47 to 0.58, and the income share of the top 1% in the US increased from 10% to 19%. Over the same period, income inequality also increased in most western European countries, including the UK, Germany and Italy, while remaining lower than in the US (Chancel 2021).

**Table 1:** Changes in Inequality from 1980 to 2019: Selected Regions

Indicators	China	East Asia	South Asia	Latin America	SSA	East Europe	West Europe	US
Gini (1980)	**0.38**	0.64	0.56	0.67	0.71	0.38	0.42	0.47
Gini (2000)	**0.50**	0.63	0.56	0.67	0.69	0.48	0.45	0.56
Gini (2019)	**0.56**	0.58	0.63	0.66	0.68	0.50	0.46	0.58
Gini (1980-2000)	**30.3%**	−1.8%	0.9%	0.2%	−1.8%	25.2%	7.7%	18.5%
Gini (2000-2019)	**11.5%**	−8.9%	11.5%	−2.1%	−2.6%	4.8%	2.1%	4.6%
Top 10% (1980)	**27.9%**	55.6%	45.7%	56.3%	57.9%	25.8%	29.9%	34.2%
Top 10% (2000)	**35.9%**	51.1%	46.3%	56.5%	57.9%	34.8%	33.5%	42.9%
Top 10% (2019)	**41.7%**	44.0%	55.6%	54.9%	55.8%	37.3%	34.8%	45.5%
Top 10% (1980-2000)	**28.6%**	−8.2%	1.5%	0.4%	0.0%	35.1%	12.0%	25.3%
Top 10% (2000-2019)	**16.1%**	−13.8%	20.0%	−2.9%	−3.7%	7.2%	3.8%	6.1%
Top 1% (1980)	**6.6%**	18.7%	20.4%	24.3%	20.8%	5.1%	8.4%	10.5%
Top 1% (2000)	**10.5%**	17.8%	18.6%	23.6%	21.3%	10.8%	10.5%	17.4%
Top 1% (2019)	**14.0%**	15.4%	21.0%	23.7%	20.4%	13.3%	11.3%	18.8%
Top 1% (1980-2000)	**59.2%**	−5.0%	−9.1%	−2.9%	2.3%	112.0%	25.2%	65.7%
Top 1% (2000-2019)	**33.8%**	−13.7%	13.2%	0.5%	−4.0%	23.1%	6.9%	8.0%

Source: World Inequality Database, https://wid.world/world/; retrieved on October 11, 2021

Developing countries’ performance varied during these four decades. Income inequality, measured by the Gini coefficient and the income shares of the top 10% and 1%, increased modestly in South Asia and East Europe. These indicators also declined slightly in Latin America and Sub-Saharan Africa. However, the latter two regions still have the highest levels of inequality.

East Asia is the only region that experienced a modest decline in regionwise income inequality. However, performance in this area varies across different countries. The Gini coefficient decreased in four countries (Malaysia, Vietnam, the Philippines and Thailand) and increased in the other countries/economies. The trend is similar when income inequality is measured in terms of the income share of the top 10% and top 1% of income groups. In particular, China’s Gini coefficient increased from 0.38 to 0.56, among the highest in Asian developing economies.

When we divide the four decades into two periods, income inequality in Western countries increased much slower during the second period (between 2000 and 2019) than during the first (between 1980 and 2000). In the US, the Gini coefficient increased by 18.5% during the two decades prior to 2000, four times that of the 20 years since 2000. The income share of the top 1% increased by 66% between 1980 and 2000, while it has only increase 8% since then. In the developing world, regional inequality also grew slower or even decreased during the first period except in South Asia, where income disparity accelerated. Overall, it appears that despite the widespread growth of inequality for the majority of the global population, there are notable variations among developing countries. East Asia performed better than other developing regions in containing inequality.

Increasing inequality is a major concern for achieving inclusive growth, which has been a key development goal for all countries adopting the 2030 SDG agenda. Although the definitions of inclusive growth vary, it is a multidimensional concept that involves fostering sustainable growth, creating opportunities, and broadening access (Ali and Zhuang 2007, Anand et al. 2013, UNCTAD 2022). Inequality is an important indicator of inclusive growth. However, promoting inclusive growth does not necessarily imply reducing inequality in all countries at any given time.

In East Asia, a sustainable period of strong economic growth has lifted millions of people out of absolute poverty. However, the benefits of growth have not been evenly distributed. A typical way of measuring the inclusiveness of growth is to determine whether the incomes of poor people increase faster than those of a population as a whole. To compare inclusive growth performance across regions, we use the World Bank indicator of the shared prosperity premium, which captures the relative performance of the bottom 40% of the population (World Bank 2020). A positive share prosperity premium indicates more inclusive growth. As shown in Table 2, nearly 80% of East Asian and Latin American economies demonstrated a trend of inclusive growth during the period 2013 to 2018, the most recent available data. However, only 58% of high-income economies (14 out of 24) have achieved inclusive growth. The proportion is only higher than in Sub-Saharan Africa (44%).

**Table 2:** Inclusive growth performance

Index	High income	East Europe & Central Asia	Latin America	Sub-Saharan Africa	East Asia	China
Annualized income growth of bottom 40%	2.23	4.46	2.00	0.67	4.30	8.38
Annualized income growth of entire population	1.87	3.58	1.18	0.27	3.56	7.12
Proportion of economies with positive shared prosperity premium	58% (14/24)	69% (18/26)	79% (11/14)	44% (4/9)	78% (7/9)	

Source: World Bank. 2021. “Global Database of Shared Prosperity and Median Income/Consumption, circa 2013-2018”, Available at https://www.worldbank.org/en/topic/poverty/brief/global-database-of-shared-prosperity. Accessed on April 4, 2022

## Retrenched welfare state

Why did Western countries seem to have a disappointing performance in containing inequality and achieving inclusive growth despite their more generous welfare states?

First, while globalization has shifted the balance between capital and labor in favor of the former, this shift is particularly pronounced in developed countries. The expansion of the global value chain and financialization has empowered MNCs, particularly certain large, “superstar firms”. As a consequence, the distributional effect of globalization is even more unbalanced, with the gain concentrated in a small number of superstar workers, superstar firms, and superstar cities (Flaherty and Rogowski 2021). In addition, heightened competition between countries further increases capital’s bargaining power, leading to a race to the bottom in national regulations and tax rates (Rudra 2008). Between 2000 and 2021, the average statutory corporate income tax rate in all OECD countries decreased by nearly 10 percentage points (OECD 2021).

Second, globalization has weakened state capacity to maintain targeted social protections to compensate distributional losers and institute effective industrial policies (Chang and Andreoni 2020). Embedded liberalism is based on the assumption that distributional losers are essentially class-based coalitions that share similar preferences with respect to protection. Compensating them with targeted protection or transfer payments is imperative for a country to engage in globalization. With multidimensional inequality and multiple political equilibriums, however, it is difficult to compensate the distributional losers and obtain their support for globalization. Moreover, the deep engagement of national economies in GVCs increases the costs for the government to provide targeted protection for low-skilled workers. If such workers are employed by companies that also fragment production in developing countries, those companies will have an incentive to oppose raising trade barriers to protect domestic low-skilled workers because of the concern that retaliation by developing countries would affect their businesses (Mansfield and Rudra 2021).

Increasing trade exposure has exerted downward pressure on welfare expenditures in developed countries (Garrett and Mitchell 2001). The growth of public social spending has been much lower than during the 1960s and 1970s as many countries have faced stronger budget constraints due to slow economic growth. Since the welfare state has increasingly become a luxury in OECD countries, they have had to tighten eligibility criteria to access social support and privatized social spending by increasing the responsibility of employers (OECD 2020). Since the global financial crisis of 2008, fear of welfare retrenchment has spurred anti-globalization demand in certain OECD countries, thus halting the declining trend of redistribution through taxes and transfers (Burgoon and Schakel 2021).

Third, increasing political polarization places substantial stress on the compromise philosophy of embedded liberalism. Many Western countries have experienced political polarization and fragmentation as they have shifted from “class-based” to “multidimensional” or “multiconflictual” party systems, in which income and education differentially structure support for competing political movements (Gethin et al. 2022). From the political perspective, demand for redistribution has been unleashed by the rise of populism and the expansion of political voices, enfranchising middle- and lower-income groups as well as women. Political elites and mass publics have become increasingly divided over public policy, ideology and ultimately partisan attachments. Rodrik argues that economic anxiety and distributional struggles exacerbated by increasing inequality generate a base for political polarization but do not necessarily determine its orientation. Populists who emphasize identity cleavage target foreigners or minorities, which produces right-wing populism. Those who emphasize the income cleavage target wealthy and large corporations, producing left-wing populism (Rodrik 2018). The latter have played a key role in generating polarization along social boundaries and restructuring political cleavage. The heightened political cleavage has brought redistributive issues to the center of political discourse.

In short, the welfare state has long been the primary approach for Western countries to pursue equitable development. Since the 1990s, welfare states have faced combined challenges, with economic stagnation and demographic changes likely to widen the gap between welfare demand and supply. Stressed social protection programs and increasing political polarization have undermined the foundation of embedded liberalism. As a consequence, Western countries have witnessed the rise of trade protectionism and populism since the 2008 global financial crisis. The COVID-19 pandemic has further exposed the vulnerability of the global interdependence of production networks, reinforcing the trend of “slowbalization” (The Economist 2019). Although welfare states have evolved in response to changes in economic and social circumstances, their essential components (i.e., high benefit levels, comprehensive social protection, and redistribution) are increasingly linked to their potential to promote inclusive growth.

## Resilient developmental state

East Asian economies’ relatively low inequality in the 20th century was the outcome of the trickle-down effect of economic growth via export orientation, investment in education, and sound economic governance, not necessarily the result of deliberate redistribution. Despite the expansion of social entitlements after democratic transition in certain East Asian economies, concerns have arisen regarding how East Asia can sustain inclusive growth in the twenty-first century, when its economic growth has been much slower than during the 1980s and 1990s.

The increasing inequality requires the state to employ more interventionist policy tools to achieve redistribution. However, the original developmental state was constrained by the transformation of the international environment, including binding trade commitments, pressure for liberalization, and the growth of international production networks. Although East Asia’s performance in poverty reduction has been much better than that of any other developing region, reliance on the trickle-down effect of growth seems to have left Asian governments ill-equipped to respond to social disruption triggered by the Asian financial crisis of 1997-98 (Haggard 2000).

Contrary to much of the literature on globalization, East Asian economies are adapting under the shadow of globalization and have retained considerable capacity to implement social programs to protect distributional losers and contain inequality. However, their performances vary depending on the dynamics of political cleavages and on social policy choices.

First, the impacts of globalization have taken different forms in East Asia. The Stopler-Samuelson theorem predicts that globalization should lead to a relative increase in the return to a country’s abundant factor, which implies that low-skilled workers in developing countries should benefit more from globalization than their counterparts in developed countries. East Asian economies are generally regarded as the winners of globalization, as most of them have benefitted significantly from export-oriented industrialization and faced less pressure from import competition. However, technological changes have a similar effect on skill premiums in both developed and developing countries. Low-skilled workers in developing countries have also experienced distributional losses because of increasing skill premiums. Therefore, while East Asian economies have also witnessed a deteriorating degree of inequality, external shocks are less salient there than what developed countries have experienced.

Second, the absence of political polarization enables the state to adopt coordinated responses to MNCs, mitigating the consequences of a race to the bottom. In developing countries, grievances driven by economic stagnation and high inequality have become sources of political polarization and antidemocratic political appeals (Haggard and Kaufman 2020). In East Asia, however, cultural values and social structure seem to continue to shape party systems in East Asia. Democratic transition has not undermined policy-making authority (Wong 2004). Instead, political participation has even declined as voters lose interest in the democratic process. The lack of political mobilization in the working class for historical reasons has played a key role in explaining the near absence of class cleavages in Japan, South Korea, and Chinese Taiwan (de la Sota and Gethin 2021).

Since the 1990s, while East Asian economies have moved in a neoliberal direction, they have retained the developmental mindset and interventionist bureaucracy in implementing industrial and social policies (Wade 2018). They have experienced substantial organizational restructuring in the pursuit of national innovative objectives. Supply-side interventions (i.e., subsidies and protection against performance conditions) are likely to privilege new products, skilled workers, and innovative business owners.

Third, East Asian economies have a more flexible and balanced social welfare structure with productivist features, including robust investment in education, relative labor flexibility, and low public social expenditure (Hudson et al. 2014). While Asian economies with favorable economic circumstances have expanded existing systems of social insurance, governments have not turned social welfare into a major source of fiscal drain (Haggard and Kaufman 2008). Social protection coverage improved from 47.1% of intended beneficiaries in 2009 to 57.3% in 2015 (Asian Development Bank 2019). Average social protection expenditure as a share of GDP has experienced modest growth, increasing from just under 4.0% in 2003–2005 to 5.3% in 2015. Even compared with other middle-income regions, East Asia has fewer and less effective social programs (World Bank 2018).

Despite expanded social protection, East Asian economies have increased the share of tax revenues in GDP in recent years, although they rely much less on taxation for redistribution than Western economies. Taxes and transfers reduced the mean Gini coefficient by 33.2% for OECD countries, while the reduction was only 6.3% for developing Asia in 2015 (Asian Development Bank 2020).

The East Asian experience of inclusive growth indicates that the developmental state is quite resilient and able to pursue adaptive reforms amidst a changing landscape of economic pressure and political cleavage. While favorable economic conditions have enabled East Asian economies to expand their redistributive capacity with mild fiscal consequences, their social policies were designed as an integral part of economic development. Thus, these policies maintain strong productivity-enhancing elements (e.g., education and health) that have been implemented through various means to stabilize employment and sustain growth.

## China’s approach to inclusive growth

The development experiences of both Western and East Asian economies suggest that achieving inclusive growth should take into account the nexus of growth, poverty and inequality. Widening inequality may negatively impact growth and poverty reduction. China appears to be an outlier with respect to this trilemma. China’s rapid economic growth since the 1980s has been accompanied by massive poverty reduction and rapidly increasing inequality.

On the one hand, China has made remarkable achievements in poverty alleviation. Its poverty rate decreased from 88% in 1981 to essentially zero by 2020, lifting approximately 800 million people out of absolute poverty. On the other, income inequality increased rapidly until the late 2000s. During the period 1980-2000, income inequality increased significantly according to various measures. The Gini coefficient, top 1% share, and top 10% share grew by 30%, 59%, and 29%, respectively. During the period 2000-2019, inequality continued to increase, albeit at a slower pace. The Gini coefficient, top 1% share, and top 10% share increased by 12%, 34%, and 16%, respectively, much higher than in other East Asian economies.

Nevertheless, measured by multiple indicators, the Chinese economy has demonstrated progress toward inclusive growth (Yu and Wang 2012). Particularly during the last decade, with the structural transformation and growth of the middle class, there has been strong evidence of inclusive growth in China. As shown in Table 2, between 2013 and 2018, China performed better in the area of inclusive growth than other developing regions, including other East Asian economies. The average income of the bottom 40% of the Chinese population increased by 8.4% annually, whereas the national average grew by 7.1%, indicating that the poor benefited more from growth than the rich.

The different trends in income inequality and inclusive growth in China are puzzling, particularly given the country’s low social welfare spending. Scholars tend to argue that China’s economic growth was driven by its hybrid institutional framework of political centralization and economic decentralization. The Chinese government possesses both the significant “infrastructural power” to reach into society and deliver on policy and the “authoritative power” to persuade individuals and groups to willingly obey commands (Evans and Heller 2013). However, the lack of broad-based embeddedness between the state and society also means that market forces overpower social protection. How did China manage to address the negative phase of Polanyi’s double movement?

We argue that China’s performance in inclusive growth can be attributed to three factors. First, rapid industrial growth and transformation have benefitted labor more than capital proportionally. Second, China counters economic disruptions caused by external shocks with massive infrastructure investment. Third, China relies on targeted transfers and state-mediated strategies for poverty alleviation. Overall, despite relatively low social spending, China’s efforts to balance distribution at the preproduction and production stages have made its economic development more inclusive.

## Industrial upgrading

In contrast to the premature deindustrialization trend witnessed in most developing countries, China’s industrial growth has accelerated since the 1990s. From 1990 to 2018, China’s industrial value-added increased 18-fold. China’s manufacturing workforce as a share of total employment increased from 10.3% in 1970 to 20.8% in 2015 (IMF 2018). As employment opportunities were widely available for labor-intensive manufacturing, industrial growth improved the welfare of labor-intensive workers throughout the country. The urban/rural household income gap has shrunk since the mid-2000s as a consequence of faster wage growth for unskilled and migrant workers relative to that of skilled urban workers (Naughton 2017).

Since the early 2000s, China has shifted from basic manufacturing to advanced manufacturing domains characterized by more skilled-labor and capital-intensive products and processes. Wages began to rise as the unlimited supply of low-cost labor disappeared. China’s industrial policy orientations have experienced important changes. Although China did not truly implement targeted industrial policy before 2006, it has been implementing industrial policy on a massive and unprecedented scale since then (Naughton 2021). In 2006, China’s National Medium-and Long-Term Program for Science and Technology Development (MLP) introduced an ambitious plan to transform the Chinese economy into a major center of innovation by 2020 and for the country to become a global leader in science and innovation by 2050. While focusing on “indigenous innovation”, the MLP also encourages leading multinationals to shift their R&D centers to China and engage in high-tech manufacturing in 10 prioritized industries. In 2015, China launched “Made in China 2025”, an industrial master plan that aims to turn the country into a “manufacturing superpower” by 2025.

As wages grew even higher during the 2010s, industrial upgrading appeared to have increased the labor share of GDP, an indicator that measures the relative share of labor compensation compared with the share paid to capital. Rising labor shares are often associated with reduced income inequality because capital income disproportionately benefits the affluent.

**Fig. 1:**
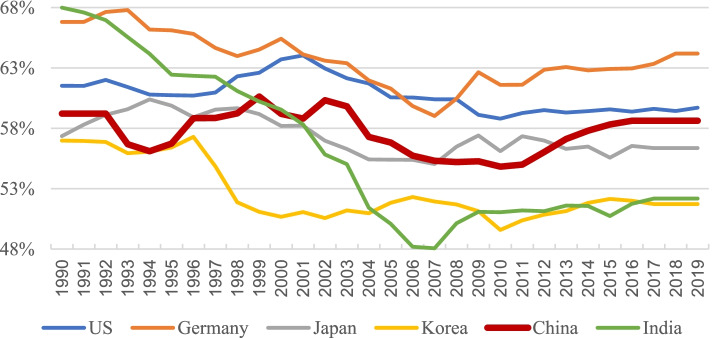
Share of Labor Compensation in GDP for Selected Countries, 1990-2019. Source: University of Groningen and University of California, Davis, Share of Labor Compensation in GDP at Current National Prices, retrieved from FRED, Federal Reserve Bank of St. Louis; https://fred.stlouisfed.org/series/LABSHPSGA156NRUG, December 1, 2021

Nevertheless, China is a notable exception to the global decline of the labor share (International Labor Organization 2019). China’s labor share has demonstrated a U-shaped pattern since 2000. It declined in the first decade of the twenty-first century and then began to rise in 2011 (Fig. 1). While China’s labor share remained lower than that of Germany and the US, it was higher than that of Japan, Korea, and India. Debate has arisen on the driving factors behind China’s rising labor share. Plausible explanations include the reduced supply of the working age population, the rise of the labor-intensive service sector (Huang and Lardy 2016), the higher growth of wages than productivity (Maarek and Orgiazzi 2020), and the polarization of formal skill-intensive industries and informal labor-intensive ones (Rozelle et al. 2020).

## Prioritizing infrastructure investment

The second driver of inclusive growth is to prioritize infrastructure investment, which is essential for economic growth and poverty reduction. This is because infrastructure investment can help increase economic activities and jobs, reduce production and transportation costs, improve access to facilities and social services, and promote technological progress (Asian Development Bank 2012).

The varying performance in infrastructure development in developing countries largely depends on governments’ investment priorities and financial capacity. While infrastructure spending in low and lower-middle income countries has declined over time, developing Asia has consistently invested a much higher share of GDP in infrastructure than other regions in the world have (Asian Development Bank 2017). In particular, China’s infrastructure spending has historically been a key driver of economic growth. The nation spent 6.31% of GDP on infrastructure spending in 2011, which was much higher than in other comparable developing countries. Even in East Asia and the Pacific region, the average level of infrastructure spending without China was 3.51% of GDP, more than two percentage points lower (Table 3).

**Table 3:** Estimates of Infrastructure Spending by Region (Central estimate), 2011

Region	Infrastructure spending (% of GDP)
East Asia and Pacific	5.72
East Europe and Central Asia	2.74
Latin America and the Caribbean	2.42
Middle East and North Africa	4.80
South Asia	4.49
Sub-Saharan Africa	1.91
LMIC weighted average	4.12

Source: Marianne Fay et al. 2019. Hitting the Trillion Mark: A Look at How Much Countries Are Spending on Infrastructure, *World Bank Policy Research Working Paper*, No. 8730 (Marianne Fay et al. 2019)

In developing countries, approximately 90% of infrastructure spending is conducted by the public sector. In nearly all countries, the share of public wealth in national wealth has declined sharply over the last two decades, particularly in developed countries. Major powers, such as the US, Japan, the UK, France, and India, even have negative public wealth due to their rapidly increasing public debt. The share of public wealth in China’s national wealth has also decreased from 41% in 2000 to 29% in 2020, and it still maintains a much higher level than most developed and developing countries (World Inequality Report 2022). Massive public wealth enables China to invest heavily in infrastructure projects (Table 4).

**Table 4:** Public wealth as a percentage of national wealth

Country	2000	2020
**China**	**41%**	**29%**
India	4%	−7%
Brazil	29%	13%
Russia	23%	19%
US	5%	−11%
Japan	13%	−5%
UK	1%	−18%
France	8%	−2%
Germany	9%	3%

Source: World Inequality Report 2022, Fig. 3.4. Available at https://wir2022.wid.world/chapter-3/; accessed on April 4, 2022

More importantly, China has relied on infrastructure investment to counter economic downturns and external shocks. For example, in the wake of the 2008 global financial crisis, the Chinese government adopted a large-scale stimulus program to bring the economy back onto a high-growth path. While the initial package amounted to 4 trillion yuan, the total stimulus expanded quickly, increasing to an estimated 27% of GDP (Wong 2011). In the midst of the COVID-19 pandemic, the Chinese government is once again relying on infrastructure investment to avoid an economic downturn. Planned infrastructure investment amounts to approximately US$ 2.3 trillion over the next 5 years, more than double the new infrastructure spending planned by the US during the same period (Hancock 2022).

## State-mediated strategy for poverty reduction

The third driver is the state-mediated strategy for poverty reduction. Industrial growth and infrastructure investment have lifted millions of Chinese out of poverty. However, eradicating poverty requires more than growth-oriented industrial policy. The poverty alleviation strategy combines development-oriented approaches with the expansion of social protection. This approach differs from populist responses to globalization that have emphasized ad hoc consumption-based spending that mainly targets the poor. While the Chinese government has made a strong commitment to combatting poverty and emphasizes that the entire country should be treated as a single chessboard (*quanguo yipan qi*), local governments have played a more important role in mobilizing resources to finance social protection programs given China’s decentralized fiscal system (Asian Development Bank 2021). In fact, despite the rapid expansion of social protection programs since 2000, actual welfare policy (e.g., Di Bao) varies greatly, determined by local government fiscal capacity and the legacy of occupation-based welfare regimes (Mok and Qian 2019).

The increase in social protection expenditure and coverage only represent part of the effort to reduce poverty. In 2013, the Chinese government launched a targeted poverty alleviation (TPA) campaign, shifting from area-based to household-based poverty targeting. The TPA granted more autonomy to local governments in launching new development projects for employment assistance and adjusting the poverty line to fit local conditions, indicating substantial decentralization in poverty alleviation (Zuo 2021). From 2013 to 2020, provincial and local governments spent nearly 1 trillion yuan on poverty reduction, 50% more than central government funds did (World Bank and Development Research Center of China 2022). Targeted infrastructure spending has also been part of national poverty-alleviation programs.

Several elements of China’s development experience appear characteristic of the classic developmental state: high growth driven by an interventionist state, a strong emphasis on fixed capital investment, and selective liberalization coupled with targeted industrial policies (Haggard 2018). However, China’s approach to inclusive growth is distinct from those of other East Asian economies in three respects. First, state-society relations are more unbalanced, with more direct state intervention to facilitate industrial upgrading and counter external shocks. Second, social policy is more integrated with industrial policy to better address development priorities. Third, local governments have demonstrated strong adaptive capacity in mobilizing resources and implementing poverty-alleviation programs. To an extent, this strategy resembles embedded neoliberalism. However, China’s approach to state-mediated intervention is designed to target the poorest and most vulnerable in society rather than privilege better-off, skilled workers and export-oriented business owners.

## Conclusion

In the second half of the 20th century, the welfare state and the developmental state were credited with facilitating the establishment of domestic social contracts in Western countries and East Asia’s embrace of globalization, respectively. Since 1990, increasing inequality and political polarization have placed redistributive pressure on both models. While Western countries have seen their welfare states retrenched or even crumble, East Asian economies have demonstrated resilience as they strengthened their redistributive capacity. They have developed a narrow version of compensation that focuses on responding to globalization through extended, institutionalized social protection in the realm of the welfare state (Lim and Burgoon 2020).

China is widely regarded as a successful catch-up for a latecomer economy. China’s massive poverty reduction and rapidly rising income inequality appear to challenge the conventional wisdom on the growth-poverty-inequality nexus. Given its relatively low development level, China has relied less on traditional means of redistribution, such as taxes and transfers. Instead, China has combined growth-oriented industrial policies and state-mediated targeted poverty alleviation programs. These policies have been effective in improving China’s inclusive growth performance, although concerns regarding increasing inequality and social polarization continue.

To an extent, China’s development strategy reveals a “third way” perspective on inclusive growth that differs from the approaches of Western welfare states and East Asian developmental states. When China began its economic reform in the early 1980s, its GDP per capita and poverty level were comparable to those of Sub-Saharan African countries. Thus, China’s development experience might be more comparable with latecomer economies. Developing countries worldwide face many development challenges and must prioritize policy actions to address these interconnected challenges. Inclusive growth cannot be achieved until a certain level of economic development is reached. Reaching this level will require coupling growth with mechanisms to directly tackle poverty, not necessarily through increasing social protection for the majority of the population.

## Data Availability

Not applicable.
